# Giant cell tumor of the tendon sheath extending around the patellar tendon and invading the knee joint and tibia: A case report

**DOI:** 10.3892/ol.2014.2561

**Published:** 2014-09-25

**Authors:** TSUTOMU AKAHANE, NAOYA MORI, KAZUSHIGE YOSHIDA

**Affiliations:** Department of Orthopaedic Surgery, Shinshu Ueda Medical Center, Ueda, Nagano 386-8610, Japan

**Keywords:** giant cell tumor of the tendon sheath, patellar tendon, knee joint, tibia

## Abstract

The current report presents the case of a 41-year-old male exhibiting a giant cell tumor of the tendon sheath (GCT-TS) arising from the patellar tendon sheath. Plain radiography and magnetic resonance imaging revealed a well-localized mass that wrapped around the patellar tendon, and extended from the subcutis into the infrapatellar fat pad and tibia. Following histopathological determination of the diagnosis, a piecemeal resection was performed. Nodular-type GCT-TS occurs less frequently in large joints compared with the small joints of the fingers and toes. The current report presents the unique features of a case of GCT-TS extending around the patellar tendon, and invading into the knee joint and proximal tibia bone.

## Introduction

Giant cell tumor of the tendon sheath (GCT-TS) is a benign soft tissue tumor of the tendon sheath and synovium (1). GCT-TS is the second most common type of tumor of the hand, and gnalgion cysts are the most common (2). The majority of GCT-TS cases occur in the fingers and toes, however, rare cases of GCT-TS occur in the knee, exhibiting a nodular pattern of growth (2). GCT-TS usually occurs in individuals between the ages of 30 and 50 years, with a predominance for females, and exhibit the capacity for local recurrence following surgical resection (1). Marginal excision is the standard treatment for of GCT-TS. However, despite its benign character, the rate of local recurrence following excision has been reported to range between 10 and 20% (1). The high local recurrence rate may be due to the fact that complete excision may be difficult, as the mass is frequently associated with the tendon sheath or synovial joint. The current report presents an unusual case of GCT-TS arising from the patellar tendon sheath, extending into the knee joint and involving the tibia. Information provided by the present report may assist clinicians in establishing a more informed diagnosis and administering the correct treatment. Written informed consent was obtained from the patient.

## Case report

In December 2012, a 41-year-old male with a 15-year history of a slow-growing, painless mass in the left knee and no history of trauma was referred to Shinshu Ueda Medical Center (Ueda, Japan) by the patient’s doctor.

**Figure 1:**
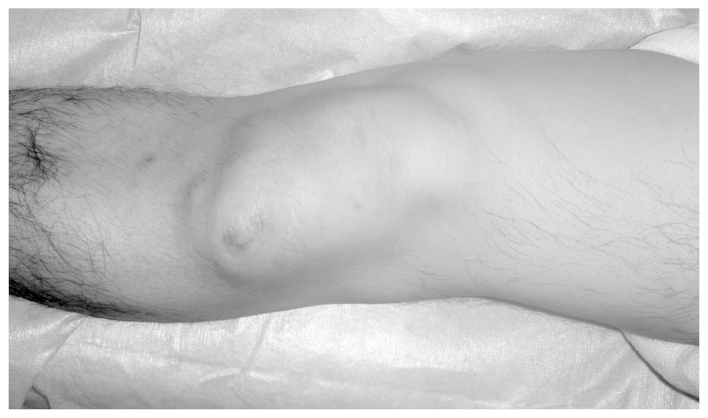
Clinical appearance of the left knee. The tumor was evident, extending from the subcutaneous tissue to the deeper aspect of the knee joint.

Physical examination revealed a mass, 8 cm in diameter, in the anterior aspect of the left knee (Fig. 1). No localized warmth, redness or tenderness was observed in the left knee, however, there was minimal effusion. The range of motion was 0–125° degrees in the left knee compared with 0–140° in the right knee. The patient displayed normal gait and no neurovascular deficit was observed.

**Figure 2:**
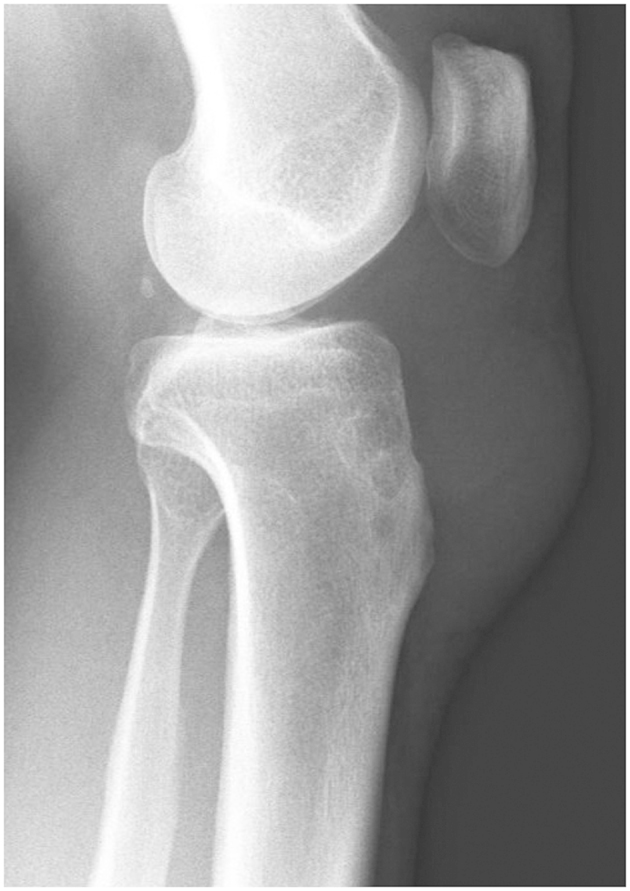
Plain lateral radiograph of the left knee demonstrating erosion of the tibia.

**Figure 3:**
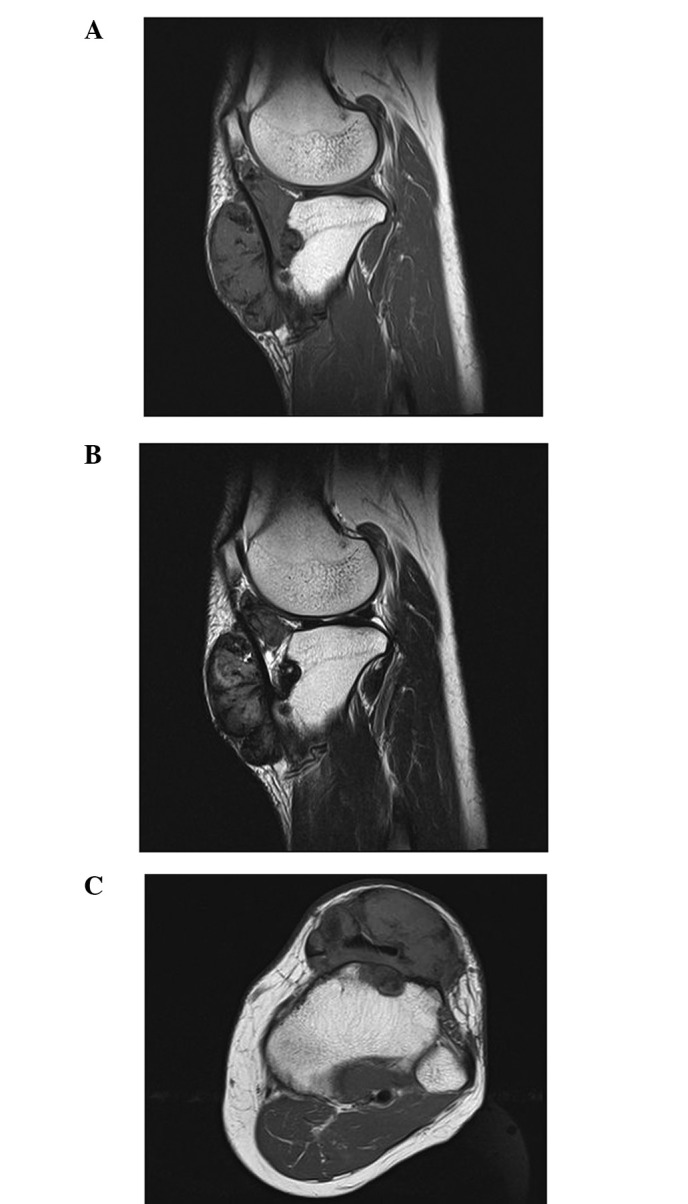
Sagittal magnetic resonance images of the left knee. (A) T1-weighted (repetition time/echo time [TR/TE]=540/12 msec) and (B) T2-weighted (TR/TE=4000/84 msec) images demonstrate a well-circumscribed soft tissue mass in the infrapatellar fat pad posterior to the patella tendon. (C) The axial image reveals a tumor wrapped around the entire circumference of the patellar tendon.

Plain lateral radiographs identified bone erosion in the proximal tibia (Fig. 2), however, no bone lesions were observed in the distal femur and patella. Magnetic resonance imaging (MRI) identified a well-localized mass that extended from the subcutaneous tissue in the anterior aspect of the knee to the infrapatellar fat pad, and deep into the patellar tendon and tibia, exhibiting a homogenous low signal intensity on T1-weighted [repetition time (TR)/echo time (TE), 540/12 msec] and T2-weighted (TR/TE, 4000/84 msec) images and diffuse enhancement (Fig. 3A and B). An axial image revealed that the tumor was wrapped around the patellar tendon (Fig. 3C).

**Figure 4:**
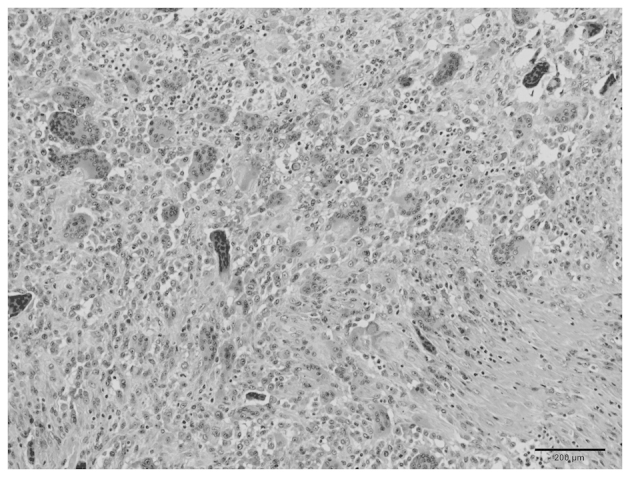
Histopathological findings in the lesion of the left knee, exhibiting a variable number of hemosiderin granules in multinucleated giant cells and sheets of mononuclear cells (hematoxylin and eosin staining; magnification, ×100).

Histopathological examination of the biopsy specimen revealed prominent histiocytes, as well as a variable number of foamy cells, hemosiderin-laden macrophages and multinucleated giant cells (Fig. 4). No mitotic activity or malignant features were observed, thus, the preoperative diagnosis was determined as GCT-TS. Although the most appropriate surgical treatment of GCT-TS is marginal resection, in the present case, en bloc marginal resection could not be achieved; therefore, a piecemeal resection was performed and the patellar tendon remained intact.

The postoperative recovery period was uneventful. The patient regained full range of motion of the knee joint and at the one-year follow-up the patient was asymptomatic. Repeat MRI scans revealed no evidence of recurrence.

## Discussion

The diffuse forms of pigmented villonodular synovitis (PVNS) and GCT-TS are classified as fibrohistiocytic tumors by the World Health Organization (3). GCT-TS occurs more frequently in the digits of the hands and feet compared with in the larger joints; whereas PVNS (also termed diffuse-type giant cell tumors) infiltrate and grow as diffuse tumors in large joints, such as the knee, elbow and ankle (3–5). While patients exhibiting diffuse-type PVNS in the knee typically present with hemarthrosis, patients exhibiting nodular-type GCT-TS typically present with a painless mass. However, certain GCT-TS in the knee joint cause continuous anterior knee pain and/or a locking knee (6,7).

The diagnosis of GCT-TS in the digits is considered to be straightforward; patients typically present with a painless mass, the lesion is usually well-circumscribed and localized, and it infrequently erodes or infiltrates adjacent bone (8). However, GCT-TS in large joints may be more difficult to diagnose, as there are few symptoms, which are non-specific (9). In such cases, differential diagnoses include synovial cyst, ganglion, synovial sarcoma, malignant fibrous histiocytoma and lipoma.

The diagnosis of GCT-TS is rarely aided by the use of plain radiographs, although, bone erosion or soft tissue swelling is occasionally observed using this imaging technique (9). In the present case, plain radiographs were used to identify tibial erosion (Fig. 2). MRI is an effective and highly sensitive tool for the diagnosis of GCT-TS and T1- and T2-weighted imaging (T1WI and T2WI) of tumors, which may present with a homogeneous low signal intensity, typically demonstrate dense collagen and hemosiderin-laden macrophages (9,10). MRI is used to determine the extent of GCT-TS by evaluating the longitudinal tumor size and the tumor extent around the phalanx (the degree of circumferential occupation by a tumor around the phalanx on an axial plane) (11). The present case demonstrated 360° tumor involvement around the patellar tendon (Fig. 3C). To the best of our knowledge, previously reported cases of nodular-type GCT-TS were not as large as the tumor investigated in the present report and exhibited less tumor involvement around the patellar tendon (6,7).

With respect to the differential diagnosis of a soft tissue mass around the knee joint, an intra-articular ganglion demonstrates a homogenous high signal intensity on T2WI; and synovial sarcoma and malignant fibrous histiocytoma exhibit a non-homogenous appearance on MRI. However, a giant cell tumor exhibits a combination of homogenous low signal intensity on T1W1 and T2WI (10). Although the present case exhibited the characteristics of a giant cell tumor on plain radiographs and MRI, histopathological determination of the diagnosis was required.

Treatment of nodular-type GCT-TS involves careful and complete local excision regardless of the site. Although GCT-TS is a benign tumor, it has a high incidence of recurrence following resection (~10–20%) (1), thus, careful observation for recurrence was required in the present case. Adequate initial local excision reduces the risk of local recurrence (5) and postoperative radiotherapy has been proposed as an optional adjuvant therapy (12).

In conclusion, the present study highlights the unique features of GCT-TS in a rare case where the tumor extended around the patellar tendon, and invaded the knee joint and tibia.

## References

[1] Weiss SW, Goldblum JR (2007). Benign tumors and tumor-like lesions of synovial tissues. Enzinger and Weiss’s Soft Tissue Tumors.

[2] Di Grazia S, Succi G, Fragetta F, Perrotta RE (2013). Giant cell tumor of tendon sheath: study of 64 cases and review of literature. G Chir.

[3] Fletcher CD, Krishnan Unni KK, Mertens F (2002). Giant cell tumour of tendon sheath. World Health Organization Classification of Tumors, Pathology and Genetics of Tumors of Soft Tissue and Bone.

[4] Ushijima M, Hashimoto H, Tsuneyoshi M, Enjoji M (1986). Giant cell tumor of the tendon sheath (nodular tenosynovitis). A study of 207 cases to compare the large joint group with the common digit group. Cancer.

[5] Monaghan H, Salter DM, Al-Nafussi A (2001). Giant cell tumour of tendon sheath (localised nodular tenosynovitis): clinicopathological features of 71 cases. J Clin Pathol.

[6] Relwani J, Factor D, Khan F, Dutta A (2003). Giant cell tumour of the patellar tendon sheath - an unusual cause of anterior knee pain: a case report. Knee.

[7] Sun C, Sheng W, Yu H, Han J (2012). Giant cell tumor of the tendon sheath: A rare case in the left knee of a 15-year-old boy. Oncol Lett.

[8] Karasick D, Karasick S (1992). Giant cell tumor of tendon sheath: spectrum of radiologic findings. Skeletal Radiol.

[9] Rodrigues C, Desai S, Chinoy R (1998). Giant cell tumor of the tendon sheath: a retrospective study of 28 cases. J Surg Oncol.

[10] Jelinek JS, Kransdorf MJ, Shmookler BM, Aboulafia AA, Malawer MM (1994). Giant cell tumor of the tendon sheath: MR findings in nine cases. AJR Am J Roentgenol.

[11] Kitagawa Y, Ito H, Amano Y, Sawaizumi T, Takeuchi T (2003). MR imaging for preoperative diagnosis and assessment of local tumor extent on localized giant cell tumor of tendon sheath. Skeletal Radiol.

[12] Kotwal PP, Gupta V, Malhotra R (2000). Giant-cell tumour of the tendon sheath. Is radiotherapy indicated to prevent recurrence after surgery?. J Bone Joint Surg Br.

